# LncRNA UCA1 facilitated cell growth and invasion through the miR-206/CLOCK axis in glioma

**DOI:** 10.1186/s12935-019-1023-7

**Published:** 2019-11-29

**Authors:** Zhi Huang, Xuya Zhao, Xiaowen Wu, Lei Xiang, Yingnan Yuan, Shi Zhou, Wenfeng Yu

**Affiliations:** 1 Department of interventional radiology, The Second Affiliated Hospital of Guizhou Medical University, Guiyang, 556000 Guizhou People’s Republic of China; 2 Department of Interventional Radiology, The Affiliated Cancer Hospital of Guizhou Medical University, Guiyang, 550005 Guizhou People’s Republic of China; 3 Key Laboratory of Endemic and Ethnic Diseases, Ministry of Education, Guizhou Medical University, No. 9 Beijing Road, Guiyang, 550002 Guizhou People’s Republic of China; 4 Department of Interventional Radiology, The Affiliated Hospital of Guizhou Medical University, No. 9 Beijing Road, Guiyang, 550002 Guizhou People’s Republic of China

**Keywords:** lncRNA UCA1, miR-206, CLOCK, Cell growth, Glioma

## Abstract

**Background:**

Glioma is a lethal malignant brain tumor, which affects the brain functions and is life-threatening. LncRNA UCA1 was identified as a pivotal regulator for tumorigenesis of glioma. MiR-206 was discovered to promote tumorigenesis and is critical in the regulation of cell proliferation in glioma. This study will discuss the expression of UCA1 regarding miR-206 and CLOCK, and their integrative effects in the proliferation and cell cycle of glioma cells.

**Methods:**

qRT-PCR was conducted to measure the mRNA expressions of IgG and Ago2 in cells co-transfected with UCA1, and miR-216 in U251. Bioinformation was analyzed for the prediction of association between UCA1 and miR-206. Transwell migrations assays and invasion assays were utilized to observe the cell invasive ability. Western blot and immunofluorescence imaging were used to examine the protein expressions. In vivo comparisons and observations were also performed to investigate the role of UCA1 in glioma growth.

**Results:**

LncRNA UCA1 was up-regulated in glioma cell lines and tissues. It elevated cell invasion via the inducing of epithelial-mesenchymal transition. We found that UCA1 can modulate miR-206 expression and serve as an endogenous sponge of miR-206. The EMT-inducer CLOCK was validated as a messenger RNA target of miR-206. At last, we demonstrated that UCA1 exerted the biology function through regulating miR-206 and CLOCK in vivo.

**Conclusions:**

Overall, the results demonstrated that UCA1/miR-206/CLOCK axis participated in the progressing of glioma and could act as a promising therapeutic target.

## Background

Glioma is a lethal malignant brain tumor, which affects the brain functions and is life-threatening [1]. It is one of the most common types of primary intracranial tumor, which comprises around 30% brain tumors, and 80% malignant brain tumors [1–3]. Researchers have paid many efforts in the study of glioma tumorigenesis to investigate appropriate treatment and accurate prognosis for glioma patients [4, 5]. Long noncoding RNAs (lncRNAs) were proved to be critical regulators in the tumorigenesis of glioma [6], such as CCAT1 [7], ZEB1-AS1 [8], TUG1 [9], and UCA1 [10]. MiRNAs were discovered to promote tumorigenesis through the targeting of some specific RNA expressions, which is critical in the regulation of cell proliferation and tumor migration in human glioma [11–13].

Previous studies have revealed that the combinational functions from lncRNA, miRNA, and the target gene, could act as a modulation axis in the regulation of solid tumors. For glioma tumors, many examples exist such as SNA1/miR-128/SP1 [14], miR-384/PIWIL4/STAT3 [15], HLF/miR-132/TTK [16]. UCA1, urothelial carcinoma-associated 1, is a lncRNA firstly cloned from the bladder cancer, which was latterly discovered as a proto-oncogene in the development of many human tumors like ovarian cancer [17], breast tumor [18], non-small cell lung cancer [19], and also glioma [20]. UCA1 could promote the proliferation and cell cycle of glioma cells via the up-regulating of cyclin D1 transcription [21].

Previous reports have identified miR-206 as an irregularly expressed gene in sodium arsenite-induced neural tube defects in chick embryos [22]. It participated in many kinds of biological activities, including skeletal muscle growth and cell tumorigenesis [23]. Its expression was down-regulated in human breast cancer [24], gastric cancer [25], and laryngeal cancer [26]. It was reported that miRNA-206 inhibited the progression of glioblastoma through BCL-2 [27]. However, the functions of miR-206 for the molecular biology in glioma remain elusive. Core circadian clock gene is an essential link between the circadian clock and human health. CLOCK heterodimers activate transcription of many proteins of the circadian clock. It is found in many tissues such as prostate, ovary, colon, and heart. Notably, it is expressed in all brain regions with the highest levels in cerebellum and plays central roles in the genesis and progression of a wide range of disorders [28, 29].

The dysregulation of lncRNA could affect microRNA expression, which could cause noticeable changes in circadian timing and output. The previous study illustrated that UCA1 acted as a sponge of miR-206 and promoted cervical cancer cell proliferation, migration, and invasion [30]. However, the expression and biological activities of UCA1 in its association with miR-206 and other related RNAs (CLOCK), especially the combinational axis in the functions of glioma, are not fully known. Therefore, therapeutic approaches designed to target the interactions and associations among the UCA1/miR-206/CLOCK attract our interest in treating glioma. This study will discuss the expression of UCA1 regarding miR-206 and CLOCK, and their integrative effects on the progressing and cell cycle of glioma.

In this research, we conducted qRT-PCR to observe the mRNA expression, transwell migrations assays and invasion assays for the cell invasive ability, western blot and immunofluorescence imaging for the protein expression, as well as in vivo comparisons and observations. Our new findings may serve as a useful prognostic indicator and an original therapeutic method for glioma.

## Materials and methods

### Cell culture and tissue samples

Glioma cell lines of U251, U87, SW1783, and LN229, and normal human astrocytes (NHAs) were obtained from Shanghai Bank of Tissues (Shanghai, China). All the cells were maintained in DMEM (Gibco, USA) with 10% FBS (Gibco, USA). They were kept at 37 °C with 5% CO_2_. Sixty groups of glioma tissues and adjacent normal tissues were harvested from the Affiliated Hospital of Guizhou Medical University. The normal tissue was obtained from patients with fresh autopsy material (donation from individuals who died in traffic accident and confirmed to be free of any prior pathologically detectable conditions). The tissues were placed in liquid nitrogen. The patients didn’t get any chemotherapy or radiotherapy before surgery, with written informed consents for their tissues. This research was performed with approval from the Ethics Committee board from the Affiliated Hospital of Guizhou Medical University.

### qRT-PCR

qRT-PCR was employed in our experiments to detect the mRNA expressions in cells transfected with various RNAs. Total RNAs were reversely transcripted via PrimeScript RT reagent (Takara, Japan). qRT-PCR was conducted using SYBR Green Master Mix II (Takara, Japan) on ABI7300. The expression of miRNA was measured with reference to U6, and was calibrated with GAPDH. The primer sequences were listed as follows:

UCA1 (forward, 5′-ACGCTAACTGGCACCTTGTT-3′ and reverse, 5′-TGGGGATTACTGGGGTAGGG-3′), GAPDH (forward, 5′-GTCAACGGATTTGGTCTGTATT-3′ and reverse, 5′-AGTCTTCTGGGTGGCAGTGAT-3′), miR-206 (forward, 5′-CGGGCTTGTGGAATGGTAAGC-3′ and reverse, 5′-GCTTCGGCAGCACATATACTAAAAT-3′), and U6 (forward, 5′-CGCTTCACGAATTTGCGTGTCAT-3′ and reverse, 5′-ATGGAACGCTTCACGA-3′).

### Migration and invasion assay

In order to examine the invasive abilities of glioma cells transfected with related genes, we carried out migration and invasion assays. For migration assay, 10,000 cells in serum-free medium were plated into the upper chamber. Culture medium was put in the lower chamber. After 1 day’s culture, the inserts were fixed and stained by 0.1% crystal violet. The migration ability could be analyzed. For invasion assay, the upper chamber of the inserts was pre-coated with 50 mg/L matrigel. Besides that, all the other procedures were the same.

### Lentiviral vector transfection

To fully understand the impact of UCA1, we used lentiviral vector transfections. U251 cells and SW1783 cells were co-transfected to lentivirus plasmid, which were constructed with shRNA of UCA1 sequence. LV-con was the comparison group. Lentiviral vector was added with UCA1 fragment to construct LV-UCA1. MiR-206 mimic or negative control (NC) was transfected via Lipofectamine 2000 (Invitrogen, USA).

### Western blotting

Western blotting was utilized in our study to reveal the protein expressions by different RNAs. Proteins were extracted via 10% SDS gel electrophoresis and transferred to polyvinylidene fluoride membranes (Millipore, USA). Antibodies to E-cadherin (1:500, ab40772, abcam), N-cadherin (1:500, ab18203, abcam), vimentin (1:500, ab8978, abcam), CLOCK (1:500, ab201974, abcam) and GAPDH (1:500, ab-8245, abcam) were purchased from Abcam, USA. The reaction was measured by chemiluminescence (Cell Signaling Technology).

### Luciferase reporter protocol

To identify the relationship among UCA1, miR-206, and CLOCK, we carried out luciferase reporter assays. UCA1-WT or UCA1-MUT binding miR-206 were sub-cloned to pGL3-Basic vector. MiR-206 mimics were co-transfected with 10 μg pLUC-WT-UCA1 or pLUC-MUT-UCA1. CLOCK-WT or CLOCK-MUT binding miR-206 were sub-cloned to pGL3-Basic vector. MiR-206 mimics were co-transfected to pLUC-WT-CLOCK or pLUC-MUT-CLOCK.

### Immunofluorescence staining

To examine the expressions of E-cadherin, N-cadherin, and vimentin immunofluorescence assays were utilized. Cells were fixed by 4% paraformaldehyde, permeabilized by 0.2% Triton X-100, and blocked by 1.5% normal donkey serum. Primary antibodies were bond for an hour followed by another hour’s incubation with Alexa Fluor 488-linked secondary antibody. The nucleus was stained with DAPI. The fluorescence was measured by a fluorescence microscope (Carl Zeiss, Germany).

### In vivo tumorigenesis

To further confirm the effects of UCA1 in glioma, in vivo experiments were performed. Eighteen female nude BALB/c mice aged 6 weeks were recruited. In front dorsum of mice, six were injected by U251 cells (5 × 10^6^/mice), six were administered of U251 cells infected by LV-sh-UCA1 (5 × 10^6^/mice), and six were administered of U251 cells infected by LV-sh-con (5 × 10^6^/mice). We recorded the tumor size every 7 days. After 6 weeks, mice were anesthetized by cervical dislocation. This study was approved by the animal experiment ethics committee of the Affiliated Hospital of Guizhou Medical University and conducted in strict accordance with the national institutes of health guidelines for the care and use of experimental animals.

### RIP assay

RNA immunoprecipitation protocol (RIP) was conducted (Millipore, USA). Cells were lysed and incubated by human anti-Ago2 antibody, with magnetic beads (Millipore, USA) or control antibody (Millipore, USA).

### Statistical analysis

SPSS 16.0 and GraphPad Prism were utilized for statistical analysis. The median values were used as cut-off scores to discriminate between low and high expression level. Data were presented as mean ± SD of at least three groups of experiments. One-way ANOVA or Student’s t-test was utilized for comparisons. Fisher’s test was employed to measure the variations between categorical variables. p < 0.05.

## Results

### UCA1 was up-regulated in glioma tissues and cell lines

**Fig. 1:**
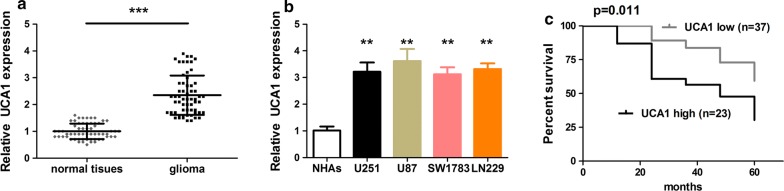
UCA1 was up-regulated in glioma tissues and cell lines. **a** Relative UCA1 expression in glioma tissues (n = 60) and normal tissues (n = 60). **b** The UCA1 expression in glioma and normal cells. **c** The Kaplan–Meier overall survival curves by UCA1 levels from 0 to 60 months. **p < 0.01, ***p < 0.001

Firstly, we aim to reveal the expression profiles of UCA1 in glioma tissues and cell lines by qRT-PCR experiments. Figure 1a displays the relative UCA1 expression in normal tissues (60) and glioma tissues (60). Compared to normal tissues, UCA1 expressions were remarkably up-regulated in glioma tissues (p < 0.001). Figure 1b shows the UCA1 expressions in U251, U87, SW1783, LN229 cells, from which UCA1 was over-expressed compared to NHAs (p < 0.01). From 0 to 60 months, Fig. 1c shows the Kaplan–Meier overall survival curves by different UCA1 levels. Long-term follow-up from this study has substantiated this finding, with survival of 86.96% at 12 months, 60.87% at 24 months, 56.52% at 36 months, 47.83% at 48 months and 30.43% at 60 months for patients with higher UCA1 levels versus 100%, 89.19%, 83.78%, 72.97% and 59.46%, respectively, for patients with lower UCA1 levels. We noticed that patients with higher UCA1 levels had poorer survival compared to those with lower levels.

### The silencing of UCA1 suppressed cell motility and invasion

**Fig. 2:**
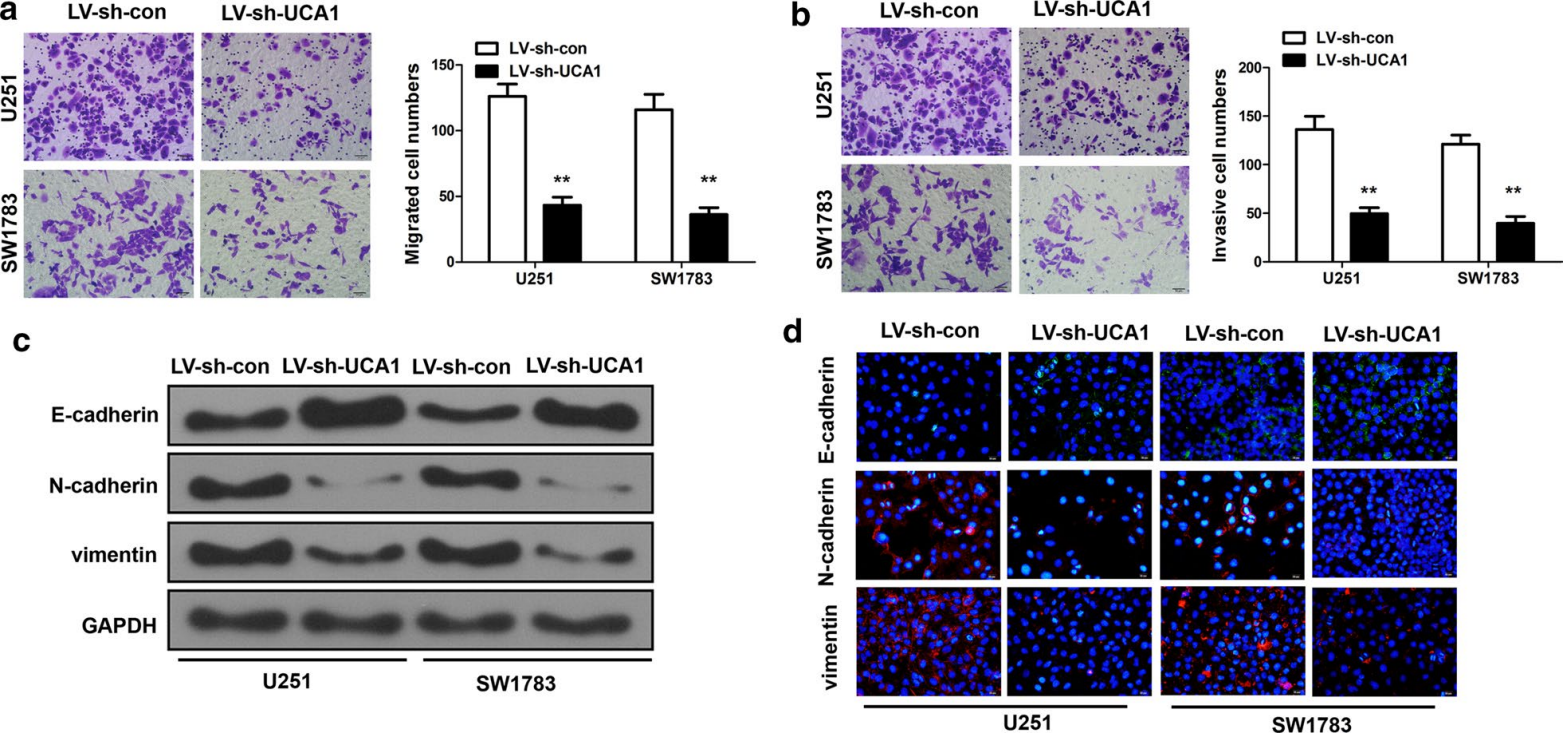
The silencing of UCA1 suppressed cell motility and invasion. **a**, **b** Transwell assay for cells transfected with LV-sh-con and LV-sh-UCA1 in U251 and SW1783. **c**, **d** The western blotting and immunofluorescence protocol for protein expressions of E-cadherin, revealed that UCA1 down-regulation increased epithelial marker E-cadherin, N-cadherin and vimentin transfected with LV-sh-con and LV-sh-UCA1 in U251 and SW1783. **p < 0.01

UCA1 was over-expressed in glioma cells and tissues. Then we hope to know the role of UCA1 in the glioma cell migration and invasion by migration assay and invasion assay. Figure 2a, b shows the transwell assay results transfected with LV-sh-con and LV-sh-UCA1 in U251 and SW1783 (p < 0.01). It revealed that the down-regulation of UCA1 inhibited glioma cell migration and invasion. Figure 2c, d demonstrates the results from western blotting and immunofluorescence. We noticed that the knockdown of UCA1 elevated the expression of epithelial marker E-cadherin, but reduced the expression of N-cadherin and vimentin.

### MiR-206 was a target of UCA1 in vitro

**Fig. 3:**
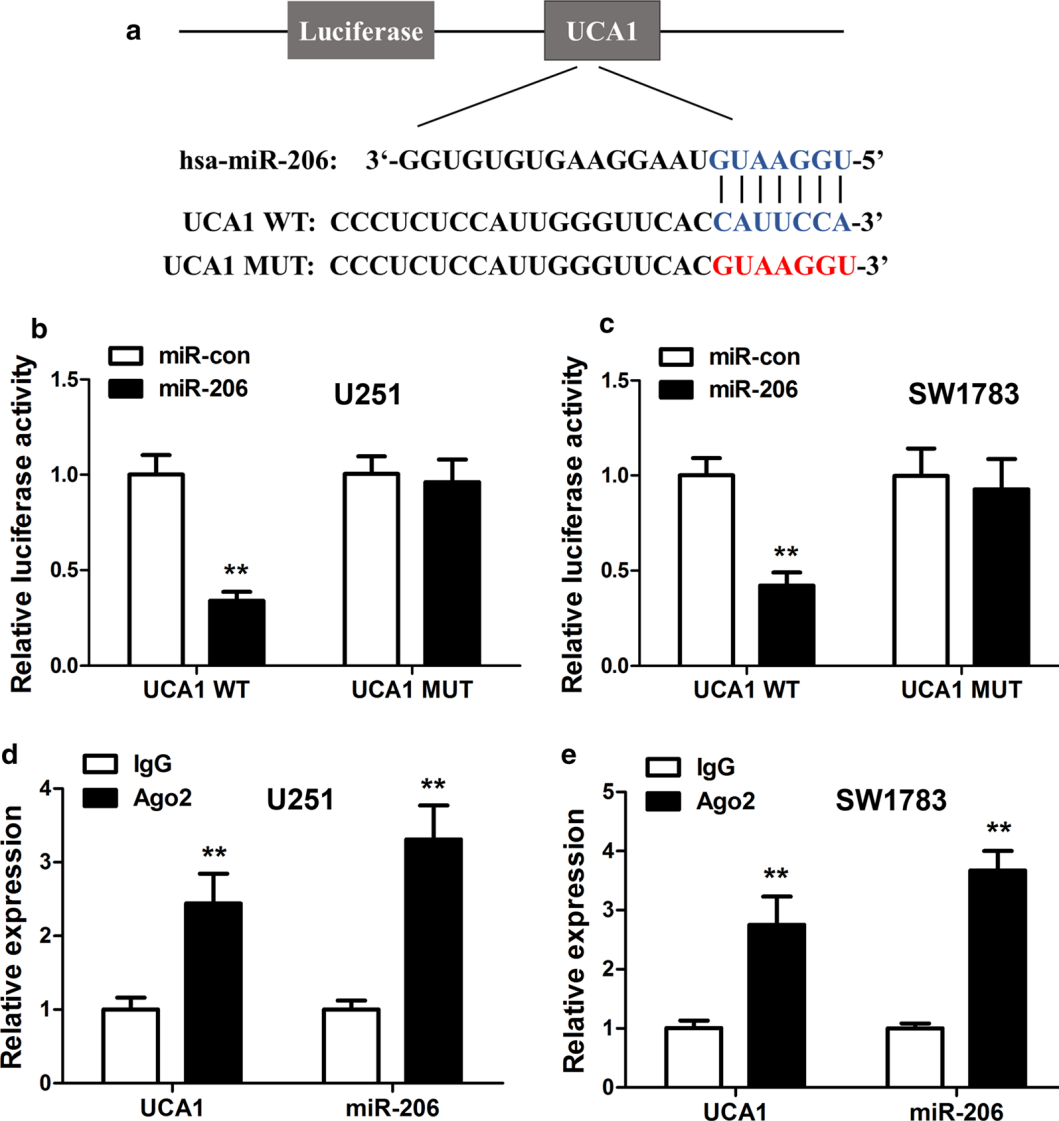
MiR-206 was a target of UCA1. **a** The common sequence between UCA1 and miR-06; and the sequence of UCA1-WT or UCA1-MUT. **b** Luciferase activities of cells transfected with miR-206 in UCA1-WT or UCA1-MUT in U251. **c** Relative luciferase activities of cells co-transfected with miR-206 in UCA1-WT or UCA1-MUT in SW1783. **d** Relative expression of IgG and Ago2 in cells co-transfected with UCA1, and miR-216 in U251. **e** Relative expression of IgG and Ago2 in cells co-transfected with UCA1, and miR-216 in SW1783. **p < 0.01

In order to understand the associations between miR-206 and UCA1, we performed online searching about their common sequences, as well as luciferase assay. Figure 3a predicts the common sequence of UCA1 and miR-206. It also shows the luciferase reporter constructs with UCA1-WT (wild-type) or UCA1-MUT (mutant). Figure 3b, c demonstrate the luciferase activity of miR-206 in UCA1-WT and UCA1-MUT, in U251 and SW1783, respectively. It was obvious that the activity of miR-206 was significantly suppressed in UCA1-WT, in both cells (p < 0.01). Figure 3c, e shows the relative expression of IgG and Ago2 in the groups of UCA1 and miR-206, in U251 and SW1783, respectively. RIP assay showed that UCA1 and miR-206 expression were more abundant in Ago2 pellet than in the control IgG pellet (p < 0.01). In addition, Additional file 1: Figure S1 also shows that miR-206 expression was highly up-regulated in cells transfected with sh-UCA1. Additional file 2: Figure S2a, c demonstrates that miR-206 expression was inhibited in glioma cancer cell lines and glioma tissues, in contrast to normal cells and tissues. From these results, the association of UCA1, miR-206, and Ago2 was confirmed, and miR-206 was a target of UCA1 in vitro.

### CLOCK served as a direct target of miR-206

**Fig. 4:**
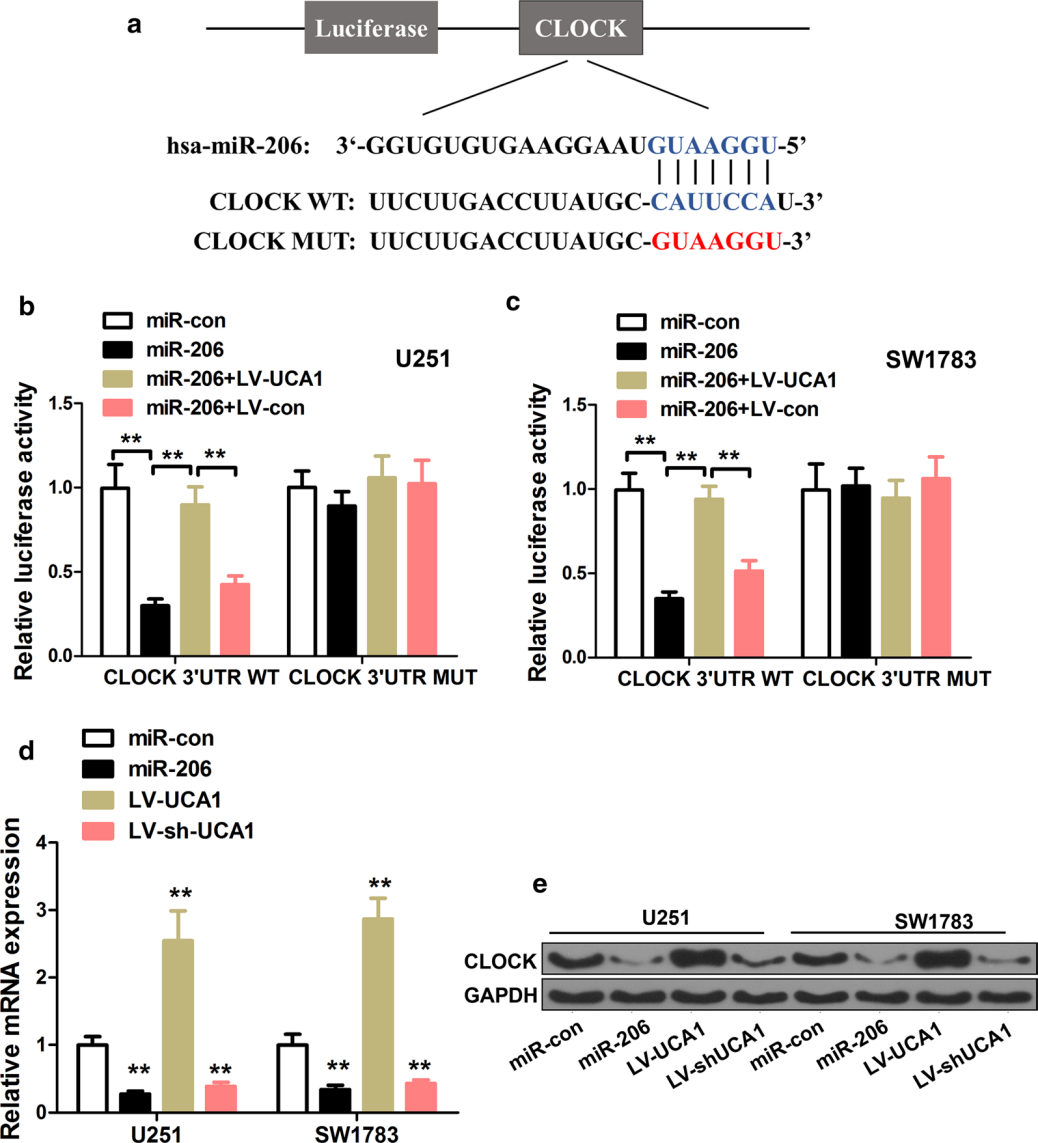
CLOCK served as a direct target of miR-206. **a** Common sequence of CLOCK and miR-206; and sequences of CLOCK-WT and CLOCK-MUT. **b** Luciferase activities with miR-206 mimics or miR-206 mimics + LV-UCA1 in U251. **c** Relative luciferase activities of cells co-transfected with miR-206 mimics or miR-206 mimics + LV-UCA1 in SW1783. **d** Relative mRNA expression of CLOCK in cells with miR-206, LV-UCA1, and LV-sh-UCA1. **e** Protein expression of CLOCK in glioma cells by western blot. **p < 0.01

Additional file 2: Figure S2b, d illustrates that CLOCK expression was up-regulated in glioma cancer cell lines and tissues, compared with normal cells and tissues. Figure 4 suggested that CLOCK was a direct target of miR-206. Figure 4a predicts the common sequence between CLOCK and miR-206. It also shows the luciferase reporter with CLOCK-WT (wild-type) and CLOCK-MUT (mutant). Figure 4b, c show the luciferase activity of CLOCK-WT or CLOCK-MUT co-transfected with miR-206 mimics or miR-206 mimics plus LV-UCA1, into U251 cells or SW1783, respectively. The luciferase activity of miR-206 was obviously reduced in CLOCK-WT, compared with CLOCK-MUT (p < 0.01). But this effect was attenuated by UCA1 (observed from the results in miR-206 + LV-UCA1). CLOCK mRNA expression was significantly decreased in glioma cells transfected with miR-206 mimics and LV-shUCA1 while it was increased in LV-UCA1 transfected glioma cells (Fig. 4d, p < 0.01). Consistent with the qRT-PCR results, western blot results indicated the same tendency (Fig. 4e, p < 0.01).

### UCA1 knockdown repressed glioma in vivo

**Fig. 5:**
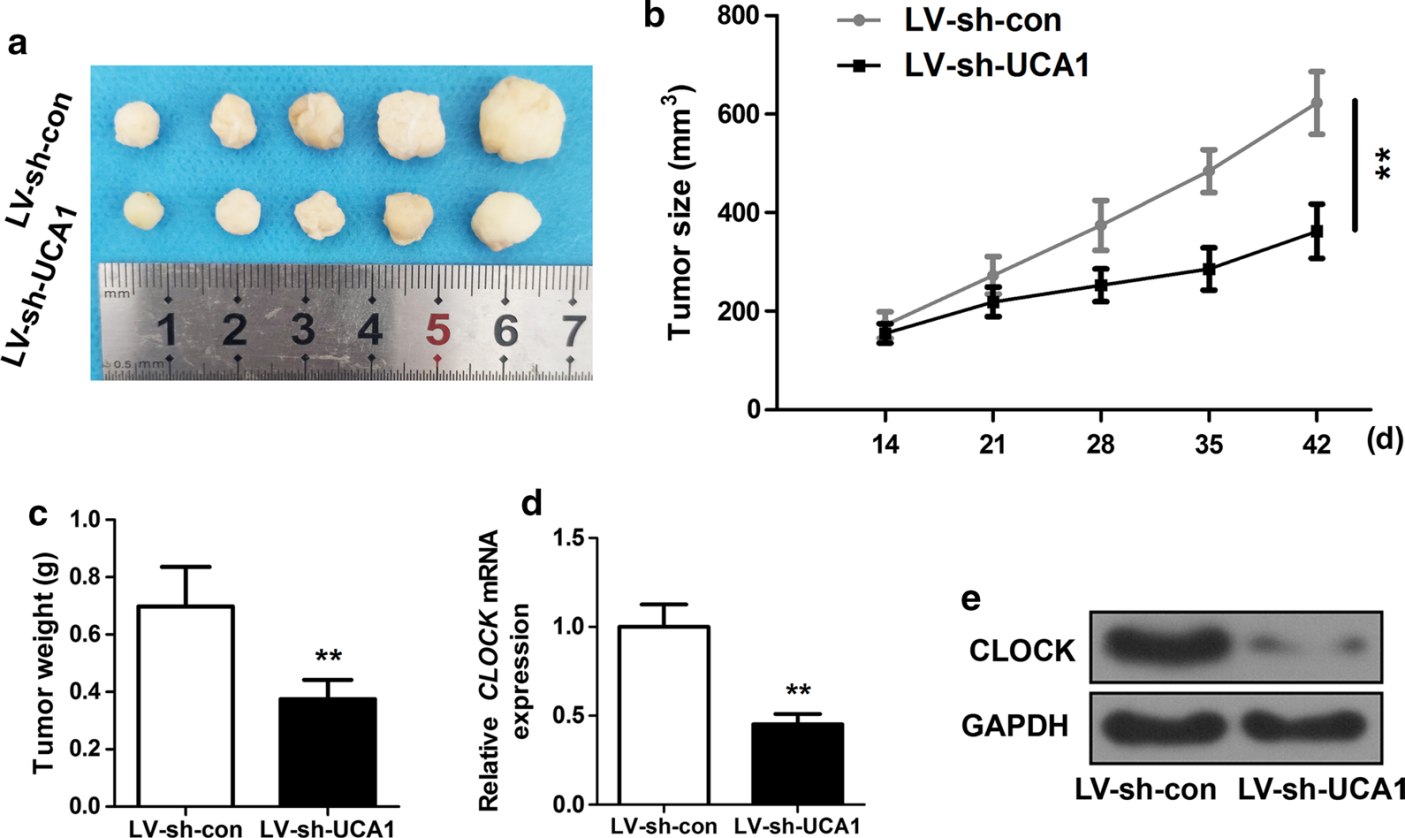
Silencing of UCA1 repressed glioma growth in vivo. **a** In vivo tumors co-transfected with LV-sh-con and LV-sh-UCA1. **b** Tumor growth curve. **c** Tumor weight. **d**, **e** CLOCK expression in LV-sh-con and LV-sh-UCA1. **p < 0.01

After characterizing the roles of UCA1, miR-206, CLOCK in glioma cells and tissues in vitro, we hope to know whether UCA1 may pose an effect on the glioma growth in vivo. Figure 5a shows the pictures of tumors in LV-sh-con and LV-sh-UCA1. Figure 5b, c displays the tumor growth curve from 0 to 42 days, and the tumor weight at 42 days. Figure 5d. e demonstrates the western blot results in LV-sh-con and LV-sh-UCA1. We noticed that CLOCK expression was suppressed by LV-sh-UCA1 in tumor tissues (p < 0.01). The data confirmed that the growth of glioma tumors was suppressed by the silencing of UCA1 in vivo. Besides, the expression of CLOCK protein was also significantly lowered by the knockdown of UCA1 (p < 0.01). In addition, we also conducted the immunohistochemistry in Additional file 3: Figure S3. We found that expression of Ki-67, a cell proliferation marker, and MMP-9, a cell metastatic marker in xenograft tumor tissue samples were inhibited in LV-sh-UCA1, in contrast with LV-sh-con (Additional file 3: Figure S3a). The same results were observed by immunohistochemistry (Additional file 3: Figure S3b). **p < 0.01. scale bar, 100 µm.

## Discussions

Glioma is a very common type of brain tumors, possessing a large portion in the malignant brain cancer which threatens the life and negatively affects the quality of the patients suffered from gliomas. Acting as a proto-oncogene, UCA1 was proved to promote the proliferation and cell cycle progression of glioma cells by upregulating cyclin D1 transcription [21]. However, minimal researchers have discovered the full associations of UCA1, miR-206, and the CLOCK gene in glioma tumor.

It has been reported that UCA1 was closely related to glioma. In 2018, Sun [10] found that UCA1 enhanced the proliferation, migration, and invasion of glioma cells via the targeting of miR-122. In their results, UCA1 expression in glioma samples was higher than the contents in normal brain samples [10]. Our qRT-PCR shows that UCA1 expression was remarkably elevated in glioma tissue and cell lines when comparing to normal tissues. Those with higher UCA1 levels had poorer overall survival than those with lower levels. This is in consistence with previous findings. He [20] demonstrated that the knockdown of UCA1 suppressed glioma cell proliferation and migration. The transwell assay, western blot, and immunofluorescence assays also indicated that the knockdown of UCA1 suppressed cell motility and invasion.

Previous researches showed that UCA1 could sponge miR-122 and promoted glioma cell progression, migration, and invasion [10]. In our study, the luciferase activity demonstrated that miR-206 could also play as a target of UCA1 in vitro. MiR-206 was discovered to be involved in many cellular activities, especially in the cell growth and tumorigenesis [31, 32]. In 2012, Wang [22] reported that miR-206 regulated the proliferation of neural cells and its apoptosis through Otx2 in glioma. In 2016, Hao [27] reported that miR-206 inhibited the progression of glioblastoma through BCL-2. In our results, the transfection of miR-206 lowered the mRNA expressions in glioma cell lines, which provides supplementary evidence of its role in the development of glioma.

Dysregulation of miR-206 expression may result in alterations in circadian timing and output [33]. Zhou [33] reported that miR-206 could mediate the dynamic mechanism of the mammalian circadian clock. The luciferase activity of CLOCK-WT co-transfected with miR-206 has indicated that CLOCK was a target of miR-206. In addition, dysregulation of the clock was reported to act as a critical role in the genesis and progression of many disorders, such as human cancers [34–36]. The western blot results in LV-sh-UCA1 revealed that CLOCK expression was suppressed in tumor tissues. This evidence indicated that the expression of CLOCK was closely related to miR-206 and UCA1, which may pose a negative effect on the development of glioma.

Limited researches have been investigated in the interactions and associations among UCA1, miR-206, and CLOCK. In 2018, He et al. [37] found that the axis of UCA1/miR-182/PFKFB2 modulated glioblastoma associated cells and the invasion of glioma. This gives us glue that multiple types of genetic markers may be considered as integration in their interaction and combinational functions in human tumors. To our best knowledge, we are the first to propose that the axis of UCA1/miR-20/CLOCK plays a critical role in the regulation of glioma development. In our experiments, CLOCK protein was less expressed in miR-206 and LV-sh-UCA1 but was significantly expressed in LV-UCA1. In addition, the in vivo tumor growth shows that UCA1 knockdown repressed glioma growth in vivo, but CLOCK expression was suppressed in tumor tissues. The growth of glioma tumors was suppressed by the silencing of UCA1 in vivo, and the expression of CLOCK protein was also significantly lowered by the knockdown of UCA1. The results demonstrated that the axis of UCA1/miR-206/CLOCK could modulate the cell proliferation of glioma cells and the growth of glioma tumors.

## Conclusions

Our experiments, results, and analysis indicated that UCA1 enhanced cell growth and invasion through the miR-206/CLOCK axis in glioma. The axis of UCA1/miR-206/CLOCK was a valid prognostic indicator and a new therapeutic method for glioma.

## Supplementary information

**Additional file 1: Figure S1.** Relative miR-206 expressions in cells transfected with LV-sh-con and LV-sh-UCA1. **p < 0.01.

**Additional file 2: Figure S2. a** miR-206 expression in glioma cancer cell lines; **b**. CLOCK expression in glioma cancer cell lines; **c**. miR-206 expression in glioma tissues; **d** CLOCK expression in glioma tissues.

**Additional file 3: Figure S3.** Expressions of Ki-67 and MMP-9 in xenograft tumor tissue samples by qPCR (**a**). Expressions of Ki-67 and MMP-9 in xenograft tumor samples by immunohistochemistry (**b**). **p < 0.01. scale bar, 100 µm.

## Data Availability

The datasets used and/or analyzed during the current study are available from the corresponding author on reasonable request.

## Abbreviations

lncRNAs: long noncoding RNAs
NHAs: normal human astrocytes
RIP: RNA immunoprecipitation protocol
