# Supplementary figures and images for: LncRNA UCA1 facilitated cell growth and invasion through the miR-206/CLOCK axis in glioma
Source: Cancer Cell Int. 2019 Nov 29;19:316. doi: 10.1186/s12935-019-1023-7 (PMC6883638; doi:10.1186/s12935-019-1023-7)

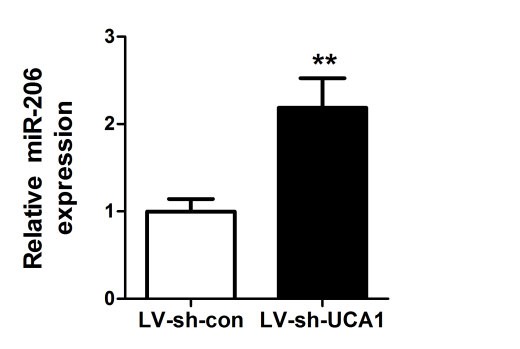

Supplement: Supplementary file 1 — Additional file 1: Figure S1. Relative miR-206 expressions in cells transfected with LV-sh-con and LV-sh-UCA1. **p < 0.01. [file 12935_2019_1023_MOESM1_ESM.tif]

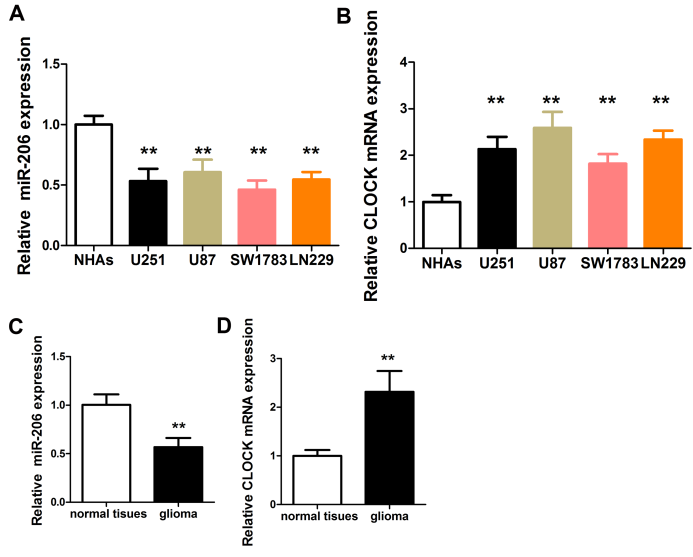

Supplement: Supplementary file 2 — Additional file 2: Figure S2. a miR-206 expression in glioma cancer cell lines; b. CLOCK expression in glioma cancer cell lines; c. miR-206 expression in glioma tissues; d CLOCK expression in glioma tissues. [file 12935_2019_1023_MOESM2_ESM.tif]

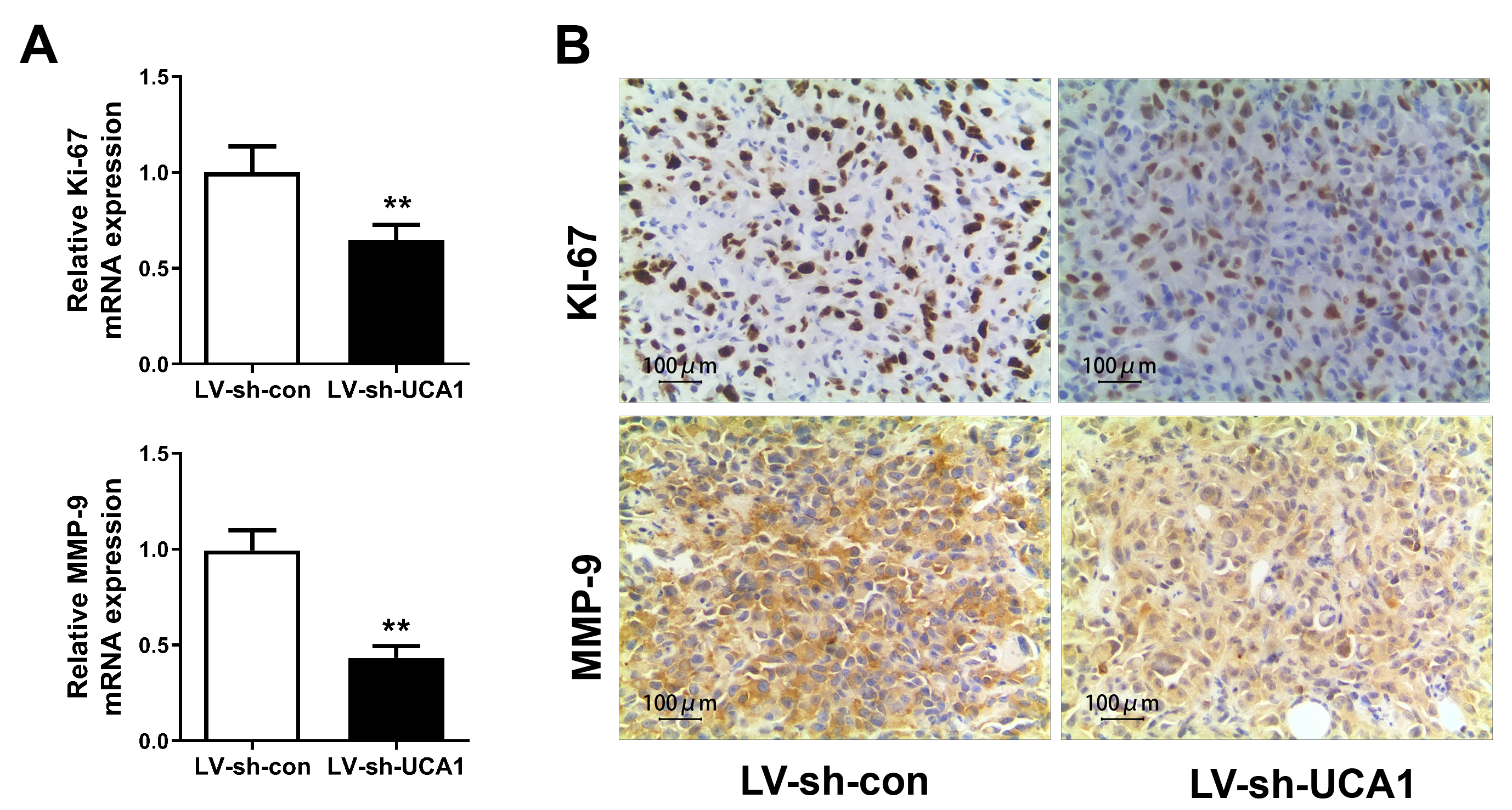

Supplement: Supplementary file 3 — Additional file 3: Figure S3. Expressions of Ki-67 and MMP-9 in xenograft tumor tissue samples by qPCR (a). Expressions of Ki-67 and MMP-9 in xenograft tumor samples by immunohistochemistry (b). **p < 0.01. scale bar, 100 µm. [file 12935_2019_1023_MOESM3_ESM.tif]
